# A model to predict participant retention in longitudinal acute pancreatitis studies

**DOI:** 10.1016/j.pan.2025.09.020

**Published:** 2025-09-22

**Authors:** Ila Lahooti, Lydia Noh, Melica Nikahd, Kathleen Tong, Anna Evans Phillips, Stacey Culp, Matthew Yoder, Patrick Rowan, Vikesh K. Singh, Elham Afghani, Ali Lahooti, Philip A. Hart, Erica Park, Somashekar G. Krishna, Raj Shah, Mitchell L. Ramsey, Georgios I. Papachristou, Peter J. Lee

**Affiliations:** a Ohio University Heritage College of Osteopathic Medicine, Dublin, OH, United States; b Northeast Ohio Medical University, Rootstown, OH, United States; c The Ohio State University Wexner Medical Center, Division of Bioinformatics, United States; d The Ohio State University College of Medicine, Columbus, OH, United States; e University of Pittsburgh Medical Center, Pittsburgh, PA, United States; f The Ohio State University Wexner Medical Center, Department of Internal Medicine, Columbus, OH, United States; g Johns Hopkins Medicine, Baltimore, MD, United States; h Weill Cornell Medical College, New York, NY, United States; i Division of Gastroenterology, Hepatology, and Nutrition, College of Medicine, The Ohio State University Wexner Medical Center, Columbus, OH, United States

**Keywords:** Longitudinal research, Follow-up, Lost to follow-up

Acute pancreatitis (AP) is an inflammatory response to pancreatic injury that is one of the leading causes of gastrointestinal hospitalizations, resulting in nearly $2.6 billion in healthcare costs each year [1]. Recently, research surrounding AP has increasingly focused on studying long-term disease outcomes [2,3]. However, participant retention is a critical challenge in longitudinal AP research, where loss to follow-up (LTFU) can compromise study validity and generalizability [4]. Thus, it is imperative to identify factors predictive of retention. Given this need, we conducted a secondary analysis of the multicenter Post-Acute Pancreatitis Pancreatic Exocrine Insufficiency (PAPPEI) study to evaluate factors predictive of participant retention, including the development of prediction models at 3- and 12-months follow-up.

Using the Revised Atlanta Criteria, the PAPPEI study enrolled adults admitted with AP across three tertiary care centers from September 2017 to September 2021. Participants completed comprehensive physical and mental baseline questionnaires, blood, and stool collection, with follow-up at 3 and 12 months. Retention was defined as completion of follow-up questionnaires and stool submission at the 3- and 12-month timepoints. Concerted efforts were made to contact participants including attempts by the research team via the participant’s communication style of choice (call, text, email), EMR messaging by the physician investigator, as well as outreach to their primary care providers. Additional details related to the study protocol have been previously described [2].

Candidate predictors collected included baseline demographic variables including age, sex, race, education, marital, and employment statuses, and income. Additionally, clinical characteristics such as alcohol-induced etiology, comorbidity burden, AP severity, and specimen submissions at baseline were considered (Supplementary Table 1). All analyses were performed using SAS 9.4. Next, two separate multivariable logistic regression models were developed for each follow-up timepoint using forward selection. Model performance was assessed with receiver operating characteristic (ROC) analysis as well as area under the ROC curve (AUC), sensitivity, specificity, and accuracy of the models. Internal validation was applied with leave out one cross-validation (LOOCV) in order to guard against model overfitting.

A total of 184 participants completed the baseline questionnaires and thus represented the baseline cohort. Of these 184 participants, ninety completed the 3-month follow-up and 100 completed the 12-month follow-up, representing retention rates of 49 % and 54 %, respectively (Supplementary Tables 2 and 3). At both follow-up timepoints, older age, higher education level, and submission of a baseline stool sample were significantly associated with higher retention. The strongest predictor of retention was baseline stool submission (OR 19.42 at 3-months; OR 7.34 at 12-months, both p < 0.001). At the 3-month timepoint, participants who were married, retired, and had a household income of at least $50,000 had statistically significant increased retention rates (Supplementary Table 2). The final 3-month model included the predictors of baseline age, education, and stool sample submission and achieved an AUC of 0.86. The 12-month model included the predictors of baseline age, stool and blood submission, education, comorbidity burden, and AP severity and had an AUC of 0.79 (Fig. 1). Internal validation was performed using LOOCV and demonstrated robust model performances. Additionally, it is important to note that Black participants were disproportionately LTFU (86 % attrition rate vs. 49 % of White participants) at 12 months.

Overall, these findings underscore the substantial attrition in longitudinal AP research despite intensive outreach efforts. The low follow-up rate among Black participants highlights a pressing need to investigate AP-specific barriers to engagement in this population [5–7]. Prior studies in other diseases of this patient population have shown that barriers include mistrust of the medical and research community, logistical and technical barriers, lack of perceived relevance and benefit, socioeconomic factors, as well as communication and engagement challenges [8]. These factors also likely contribute to attrition rates in longitudinal AP research and should serve as a foundation for future studies aimed at improving retention in this patient population.

Our study also identified the notable influence of sociodemographic factors on participant retention. Higher education levels and increased age were significantly associated with follow-up completion at both the 3- and 12-month periods, suggesting that older participants may have more flexibility and time that enable them to complete follow-ups; additionally, in a study by Kim et al., it was found that patients with higher levels of education may experience less barriers to study participation including, but not limited to, transportation, childcare, work schedules, and psychological challenges [9]. Further understanding these socioeconomic influences on retention can help inform targeted strategies, such as tailored outreach efforts and resource allocation, that may reduce barriers to participant follow-up and thereby improve retention in diverse patient populations.

Finally, our results demonstrate that an early willingness to submit a stool sample was the strongest indicator of long-term participation, suggesting patient engagement at baseline is critical. Yet, nearly 40 % of participants did not submit a stool sample, highlighting a persistent challenge in AP research retention. Prior interview-based studies have shown that patients often find stool collection inconvenient and lack confidence or adequate instruction on how to complete the process [10]. Addressing these barriers by simplifying the collection process and offering greater patient support and education is an actionable opportunity for future longitudinal AP studies to improve retention.

To our knowledge, our study is the first to develop internally validated models to predict participant retention in longitudinal AP research. A strength of our study is that our model uses an accessible formula (Supplementary Materials) with a small set of readily available variables that can easily be replicated for external application. Our study is limited by a modest sample size, due to the practical limitations of resource constraints, that precluded the evaluation of model fairness. Additionally, participants were enrolled non-consecutively, therefore our AP cohort may not be representative of the overall AP cohort. Future studies should apply this model to a larger, more representative cohort.

In conclusion, participant retention remains a major challenge in longitudinal AP studies. Our study provides an accessible prediction model to assess retention risk in patients with AP, allowing targeted interventions to maximize participant follow-up and thereby strengthen the generalizability of AP research.

## Supplementary Material

Supplementary Material

## Figures and Tables

**Fig. 1.**
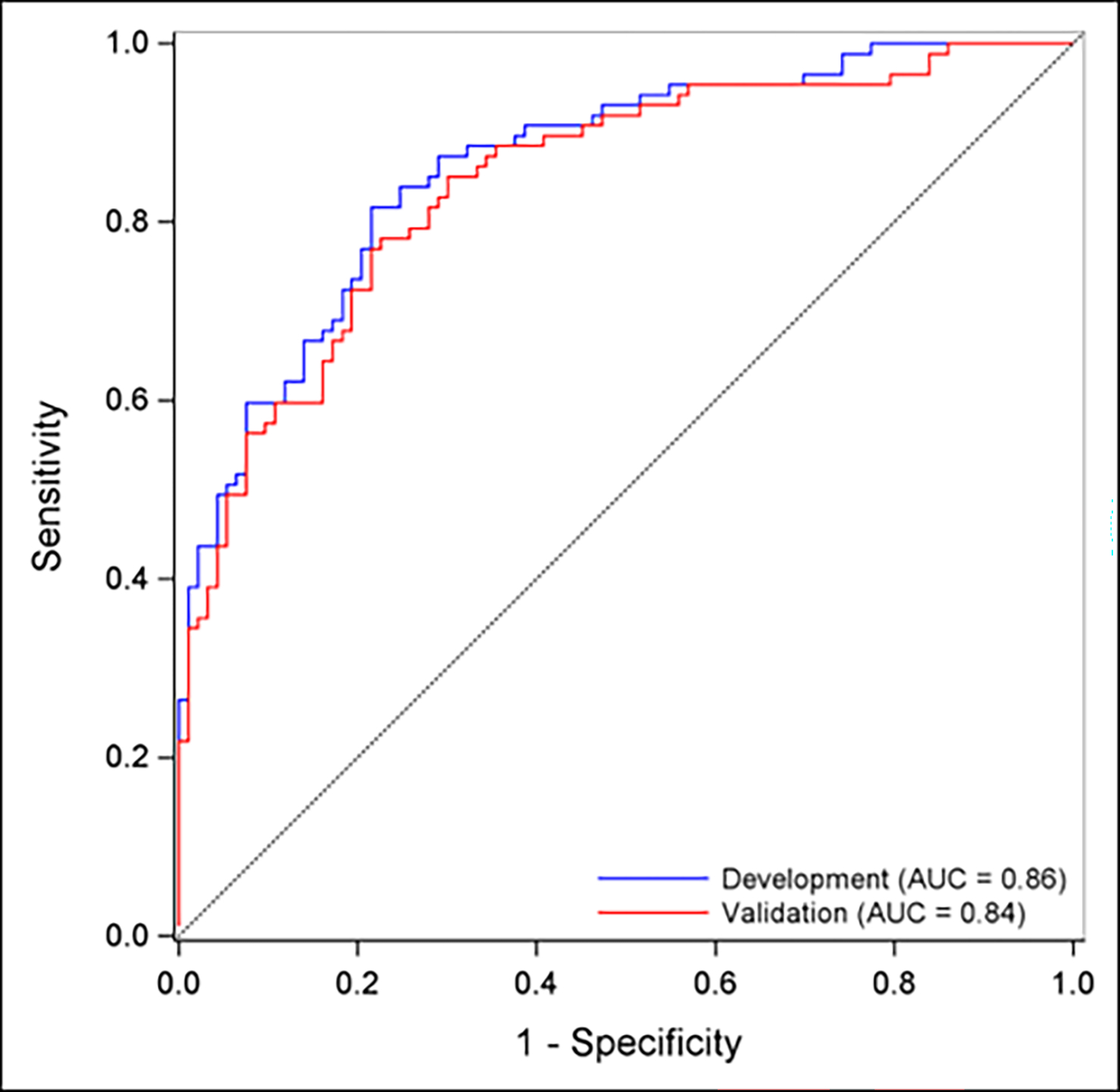

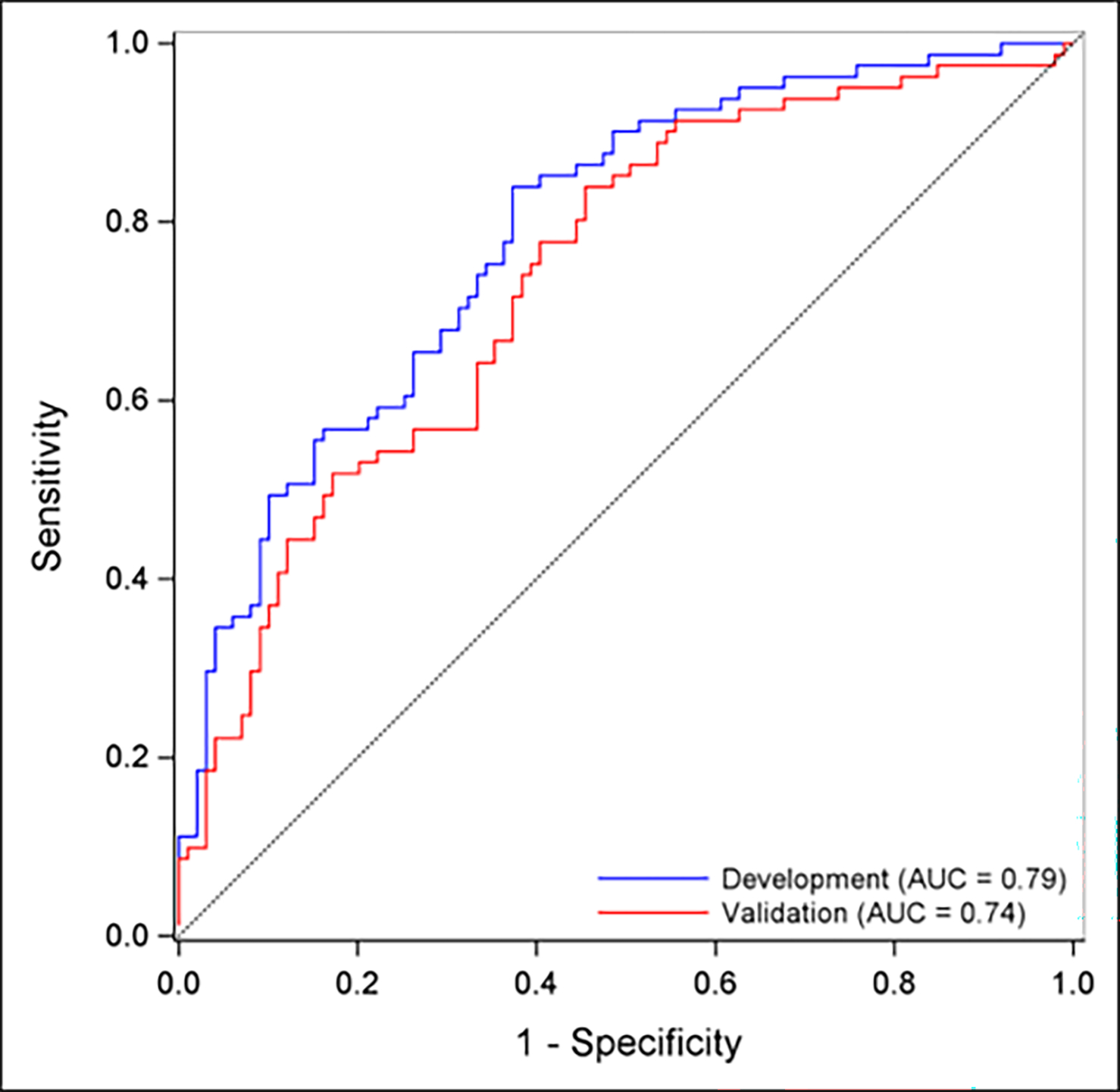
ROC curves of the developed and validation models to predict participant retention at 3 and 12 months, respectively.

## Appendix A. Supplementary data

Supplementary data to this article can be found online at https://doi.org/10.1016/j.pan.2025.09.020.
