# Supplementary material for: A model to predict participant retention in longitudinal acute pancreatitis studies
Source: Pancreatology. Author manuscript; Available in PMC 2026 Feb 18. (PMC12915571; doi:10.1016/j.pan.2025.09.020)
Supplement: Supplementary Material [file NIHMS2143986-supplement-Supplementary_Material.docx]

**Supplemental Materials:**

Supplementary Table 1. Predictors and outcomes that were recategorized or scored from survey data

| Variable | Definition/levels |
| --- | --- |
| **Outcomes** | |
| 3-month retention | Completion of 3-month questionnaires and stool sample |
| 12-month retention | Completion of 12-month questionnaires and stool sample |
| **Candidate Predictors** | |
| Race | Categorized into “white”, “black”, and “other”; “other” includes American Indian/Alaska Native, Asian, Native Hawaiian or other Pacific Islander, and unknown/refuses to select a race |
| Education | Collapsed into the following categories: “high school education or less”, “Associate’s degree/vocational school/some college”, and “Bachelor’s degree or higher” |
| Marital status | Married vs. widowed, divorced, separated, or single |
| Employment status | Collapsed into the following categories: “retired”, “employed for wages or self-employed”, “unemployed”, and “other”; “other” includes student, homemaker, and military workers |
| Annual household income | Categorized into “low” (<$50,000/year), “middle” ($50,000– $100,000/year), and “high” (>$100,000/year) |
| Activity category | Based on Godin Leisure-Time Exercise Questionnaire; categories included “insufficiently active/sedentary”, “moderately active”, and “active” |
| CCI | Non-age adjusted Charlson comorbidity index |
| Healthy Lifestyle Score | Patients were scored on a scale from 0-7; each patient received 1 point if they consumed ≤ 2 alcoholic beverages per day for men or ≤ 1 for women, never smoked, had a normal BMI (18-24.99), were active or moderately active, consumed ≥124 oz of fluids/day for men or ≥92 oz of fluids/day for women, did not often consume or never consumed red meat, did not often consume or never consumed chocolate or candy, and had more than 3 servings of fruits/vegetables per day |

Supplementary Table 2. Demographic and clinical characteristics by 3-month retention status* (N=184); bolded p-values were significant

|  | Retained  (n=90) | Not Retained  (n=94) | Total  (N=184) | p-value^†^ |
| --- | --- | --- | --- | --- |
|  | Demographics | | |  |
| Age, mean (SD) | 55.5 (13.8) | 47.2 (15.5) | 51.3 (15.2) | **<0.001** |
| Male, n (%) | 40 (44.4%) | 49 (52.1%) | 89 (48.4%) | 0.30 |
| Race, n (%) |  |  |  |  |
| White | 79 (87.8%) | 74 (78.7%) | 153 (83.2%) | 0.28 |
| Black | 8 (8.9%) | 14 (14.9%) | 22 (12.0%) |  |
| Other | 3 (3.3%) | 6 (6.4%) | 9 (4.9%) |  |
| Education, n (%) |  |  |  |  |
| High school education or less | 17 (19.5%) | 41 (44.1%) | 58 (32.2%) | **<0.001** |
| Associate’s degree/vocational school/some college | 27 (31.0%) | 29 (31.2%) | 56 (31.1%) |  |
| Bachelor’s degree or higher | 43 (49.4%) | 23 (24.7%) | 66 (36.7%) |  |
| Marital Status, n (%) |  |  |  |  |
| Widowed/divorced/  separated/single | 31 (34.8%) | 48 (52.7%) | 79 (43.9%) | **0.015** |
| Married | 58 (65.2%) | 43 (47.3%) | 101 (56.1%) |  |
| Employment Status, n (%) |  |  |  |  |
| Retired | 29 (33.7%) | 15 (16.3%) | 44 (24.7%) | **0.031** |
| Employed for wages or self-employed | 37 (43.0%) | 46 (50.0%) | 83 (46.6%) |  |
| Unemployed | 18 (20.9%) | 24 (26.1%) | 42 (23.6%) |  |
| Other | 2 (2.3%) | 7 (7.6%) | 9 (5.1%) |  |
| Household annual income, n (%) |  |  |  |  |
| <$50,000 | 27 (38.0%) | 47 (59.5%) | 74 (49.3%) | **0.030** |
| $50,000-$100,000 | 26 (36.6%) | 20 (25.3%) | 46 (30.7%) |  |
| >$100,000 | 18 (25.4%) | 12 (15.2%) | 30 (20.0%) |  |
|  | Clinical Characteristics | | |  |
| Enrolling Site, n (%) |  |  |  |  |
| Johns Hopkins | 0 (0.0%) | 7 (7.4%) | 7 (3.8%) | **0.029** |
| Ohio State University | 42 (46.7%) | 41 (43.6%) | 83 (45.1%) |  |
| University of Pittsburgh | 48 (53.3%) | 46 (48.9%) | 94 (51.1%) |  |
| BMI, mean (SD) | 31.8 (10.1) | 33.1 (11.3) | 32.4 (10.7) | 0.40 |
| ICU Admission, n (%) | 8 (9.5%) | 10 (12.2%) | 18 (10.8%) | 0.58 |
| Alcoholic Etiology, n (%) | 9 (10.0%) | 17 (18.1%) | 26 (14.1%) | 0.12 |
| History of AP, n (%) | 31 (34.4%) | 41 (43.6%) | 72 (39.1%) | 0.20 |
| Revised Atlanta Classification, n (%) |  |  |  |  |
| Mild | 55 (61.1%) | 59 (62.8%) | 114 (62.0%) | 0.70 |
| Moderately severe | 27 (30.0%) | 24 (25.5%) | 51 (27.7%) |  |
| Severe | 8 (8.9%) | 11 (11.7%) | 19 (10.3%) |  |
| Activity, n (%) |  |  |  |  |
| Insufficiently Active/Sedentary | 46 (51.1%) | 48 (51.1%) | 94 (51.1%) | 0.91 |
| Moderately Active | 23 (25.6%) | 22 (23.4%) | 45 (24.5%) |  |
| Active | 21 (23.3%) | 24 (25.5%) | 45 (24.5%) |  |
| Smoking, n (%) |  |  |  |  |
| Never (<100 cigarettes or 5 packs in lifetime) | 47 (52.8%) | 42 (44.7%) | 89 (48.6%) | 0.27 |
| Active (within the last 6 months) | 15 (16.9%) | 25 (26.6%) | 40 (21.9%) |  |
| Former (>6 months without smoking) | 27 (30.3%) | 27 (28.7%) | 54 (29.5%) |  |
| Alcohol pattern in 6 mo. before getting pancreatitis, n (%) |  |  |  |  |
| Frequent (15 days or more per month) | 9 (10%) | 15 (16%) | 24 (13%) | 0.58 |
| Occasional (less than 15 days) | 12 (13.3%) | 14 (14.9%) | 26 (14.1%) |  |
| Weekend mainly | 9 (10%) | 11 (11.7%) | 20 (10.9%) |  |
| Binge (at least 3 days heavy drinking) | 0 (0%) | 2 (2.1%) | 2 (1.1%) |  |
| Special Occasions | 17 (18.9%) | 14 (14.9%) | 31 (16.8%) |  |
| Abstinent | 18 (20%) | 14 (14.9%) | 32 (17.4%) |  |
| Not Sure | 1 (1.1%) | 1 (1.1%) | 2 (1.1%) |  |
| Not active or former drinker | 22 (24.4%) | 23 (24.5%) | 45 (24.5%) |  |
| Chronic Narcotics Use, n (%) | 4 (4.5%) | 4 (4.4%) | 8 (4.5%) | 1.00 |
| Cholecystectomy, n (%) | 17 (18.9%) | 17 (18.1%) | 34 (18.5%) | 0.89 |
| CCI, mean (SD) | 0.9 (1.4) | 1.1 (1.6) | 1.0 (1.5) | 0.47 |
| Healthy Lifestyle Score^‡^, mean (SD) | 3.7 (1.3) | 3.8 (1.1) | 3.7 (1.2) | 0.90 |
| EPI Symptom Burden, mean (SD) | 2.5 (2.6) | 3.3 (3.0) | 2.9 (2.8) | 0.08 |
| Baseline blood submitted, n (%) | 69 (76.7%) | 80 (85.1%) | 149 (81%) | 0.14 |
| Baseline stool submitted, n (%) | 79 (87.8%) | 38 (40.4%) | 117 (63.6%) | **<0.001** |

*Patients were considered “retained” at 3-month follow-up if they completed both the follow-up questionnaire and stool sample at 3-month follow-up

^‡^Patients were scored on a scale from 0-7; each patient received 1 point if they consumed ≤ 2 alcoholic beverages per day for men or ≤ 1 for women, never smoked, had a normal BMI (18-24.99), were active or moderately active, consumed ≥124 oz of fluids/day for men or ≥92 oz of fluids/day for women, did not often consume or never consumed red meat, did not often consume or never consumed chocolate or candy, and had more than 3 servings of fruits/vegetables per day.

^†^p-value from chi-square or fisher exact (when expected counts ≤ 5) test for categorical predictors and two-sample t-test for continuous predictors

Supplementary Table 3. Demographic and clinical characteristics by 12-month retention status* (N=184); bolded p-values were significant

|  | Retained  (n=100) | Not Retained  (n=84) | Total  (N=184) | p-value^†^ |
| --- | --- | --- | --- | --- |
|  | Demographics | | |  |
| Age, mean (SD) | 54.8 (14.1) | 48.3 (15.5) | 51.3 (15.2) | **0.004** |
| Male, n (%) | 43 (51.2%) | 46 (46.0%) | 89 (48.4%) | 0.48 |
| Race, n (%) |  |  |  |  |
| White | 78 (92.9%) | 75 (75.0%) | 153 (83.2%) | **0.002** |
| Black | 3 (3.6%) | 19 (19.0%) | 22 (12.0%) |  |
| Other | 3 (3.6%) | 6 (6.0%) | 9 (4.9%) |  |
| Education, n (%) |  |  |  |  |
| High school education or less | 19 (23.5%) | 39 (39.4%) | 58 (32.2%) | **0.010** |
| Associate’s degree/vocational school/some college | 23 (28.4%) | 33 (33.3%) | 56 (31.1%) |  |
| Bachelor’s degree or higher | 39 (48.1%) | 27 (27.3%) | 66 (36.7%) |  |
| Marital Status, n (%) |  |  |  |  |
| Widowed/divorced/  separated/single | 30 (36.6%) | 49 (50.0%) | 79 (43.9%) | 0.07 |
| Married | 52 (63.4%) | 49 (50.0%) | 101 (56.1%) |  |
| Employment Status, n (%) |  |  |  |  |
| Retired | 24 (30.4%) | 20 (20.2%) | 44 (24.7%) | 0.19 |
| Employed for wages or self-employed | 38 (48.1%) | 45 (45.5%) | 83 (46.6%) |  |
| Unemployed | 15 (19.0%) | 27 (27.3%) | 42 (23.6%) |  |
| Other | 2 (2.5%) | 7 (7.1%) | 9 (5.1%) |  |
| Household annual income, n (%) |  |  |  |  |
| <$50,000 | 27 (39.1%) | 47 (58.0%) | 74 (49.3%) | 0.07 |
| $50,000-$100,000 | 25 (36.2%) | 21 (25.9%) | 46 (30.7%) |  |
| >$100,000 | 17 (24.6%) | 13 (16.0%) | 30 (20.0%) |  |
|  | Clinical Characteristics | | |  |
| Enrolling Site, n (%) |  |  |  |  |
| Johns Hopkins | 0 (0.0%) | 7 (7.0%) | 7 (3.8%) | **0.029** |
| Ohio State University | 42 (50.0%) | 41 (41.0%) | 83 (45.1%) |  |
| University of Pittsburgh | 42 (50.0%) | 52 (52.0%) | 94 (51.1%) |  |
| BMI, mean (SD) | 32.3 (10.4) | 32.5 (11.1) | 32.4 (10.7) | 0.91 |
| ICU Admission, n (%) | 11 (13.8%) | 7 (8.1%) | 18 (10.8%) | 0.25 |
| Alcoholic Etiology, n (%) | 9 (10.7%) | 17 (17.0%) | 26 (14.1%) | 0.22 |
| History of AP, n (%) | 29 (34.5%) | 43 (43.0%) | 72 (39.1%) | 0.24 |
| Revised Atlanta Classification, n (%) |  |  |  |  |
| Mild | 51 (60.7%) | 63 (63.0%) | 114 (62.0%) | 0.83 |
| Moderately severe | 25 (29.8%) | 26 (26.0%) | 51 (27.7%) |  |
| Severe | 8 (9.5%) | 11 (11.0%) | 19 (10.3%) |  |
| Activity, n (%) |  |  |  |  |
| Insufficiently Active/Sedentary | 44 (52.4%) | 50 (50.0%) | 94 (51.1%) | 0.95 |
| Moderately Active | 20 (23.8%) | 25 (25.0%) | 45 (24.5%) |  |
| Active | 20 (23.8%) | 25 (25.0%) | 45 (24.5%) |  |
| Smoking, n (%) |  |  |  |  |
| Never (<100 cigarettes or 5 packs in lifetime) | 45 (54.2%) | 44 (44.0%) | 89 (48.6%) | **0.037** |
| Active (within the last 6 months) | 11 (13.3%) | 29 (29.0%) | 40 (21.9%) |  |
| Former (>6 months without smoking) | 27 (32.5%) | 27 (27.0%) | 54 (29.5%) |  |
| Alcohol pattern in 6 mo. before getting pancreatitis, n (%) |  |  |  |  |
| Frequent (15 days or more per month) | 10 (12.2%) | 14 (14.0%) | 24 (13.2%) | 0.71 |
| Occasional (less than 15 days) | 10 (12.2%) | 16 (16.0%) | 26 (14.3%) |  |
| Weekend mainly | 8 (9.8%) | 12 (12.0%) | 20 (11.0%) |  |
| Binge (at least 3 days heavy drinking) | 0 (0.0%) | 2 (2.0%) | 2 (1.1%) |  |
| Special Occasions | 12 (14.6%) | 19 (19.0%) | 31 (17.0%) |  |
| Abstinent | 17 (20.7%) | 15 (15.0%) | 32 (17.6%) |  |
| Not Sure | 1 (1.2%) | 1 (1.0%) | 2 (1.1%) |  |
| Not active or former drinker | 24 (29.3%) | 21 (21.0%) | 45 (24.7%) |  |
| Chronic Narcotics Use, n (%) | 3 (3.7%) | 5 (5.1%) | 8 (4.5%) | 0.73 |
| Cholecystectomy, n (%) | 18 (21.4%) | 16 (16.0%) | 34 (18.5%) | 0.34 |
| CCI, mean (SD) | 0.9 (1.2) | 1.1 (1.7) | 1.0 (1.5) | 0.36 |
| Healthy Lifestyle Score^‡^, mean (SD) | 3.8 (1.3) | 3.7 (1.1) | 3.7 (1.2) | 0.96 |
| EPI Symptom Burden, mean (SD) | 2.9 (2.8) | 2.9 (2.9) | 2.9 (2.8) | 0.99 |
| Baseline blood submitted, n (%) | 62 (73.8%) | 87 (87.0%) | 149 (81.0%) | **0.023** |
| Baseline stool submitted, n (%) | 70 (83.3%) | 42 (42.0%) | 112 (60.9%) | **<0.001** |

*Patients were considered “retained” at 3-month follow-up if they completed both the follow-up questionnaire and stool sample at 3-month follow-up

^‡^Patients were scored on a scale from 0-7; each patient received 1 point if they consumed ≤ 2 alcoholic beverages per day for men or ≤ 1 for women, never smoked, had a normal BMI (18-24.99), were active or moderately active, consumed ≥124 oz of fluids/day for men or ≥92 oz of fluids/day for women, did not often consume or never consumed red meat, did not often consume or never consumed chocolate or candy, and had more than 3 servings of fruits/vegetables per day.

^†^p-value from chi-square or fisher exact (when expected counts ≤ 5) test for categorical predictors and two-sample t-test for continuous predictors

Supplementary Table 4. Odds ratios, 95% confidence intervals, and p-values from multivariable prediction model assessing the odds of study retention at 3-months; bolded p-values were significant

|  | Comparison | Reference | OR (95% CI) | p-value |
| --- | --- | --- | --- | --- |
| Baseline stool sample | Collected | Not Collected | 19.42 (7.63, 49.40) | **<0.001** |
| Education | Associates degree/vocational school/some college | High school education or less | 2.22 (0.89, 5.54) | 0.09 |
|  |  |  |  |  |
|  | Bachelor’s degree or higher |  | 7.68 (2.75, 21.44) | **<0.001** |
| Baseline age | 1-year increase | | 1.03 (1.01, 1.06) | **0.017** |

Supplementary Table 5. Odds ratios, 95% confidence intervals, and p-values from multivariable prediction model assessing the odds of study retention at 12 months; bolded p-values were significant

|  | Comparison | Reference | OR (95% CI) | p-value |
| --- | --- | --- | --- | --- |
| Baseline stool sample | Collected | Not Collected | 7.34 (3.40, 15.86) | **<0.001** |
| Baseline blood sample | Collected | Not Collected | 0.45 (0.19, 1.08) | 0.07 |
| Education | Associates degree/vocational school/some college | High school education or less | 1.14 (0.48, 2.70) | 0.77 |
|  |  |  |  |  |
|  | Bachelor’s degree or higher |  | 2.99 (1.25, 7.12) | **0.014** |
| Revised Atlanta Classification | Moderately Severe AP | Mild AP | 1.38 (0.63, 3.06) | 0.42 |
|  |  |  |  |  |
|  | Severe AP |  | 0.93 (0.31, 2.84) | 0.90 |
| Baseline age | 1-year increase | | 1.02 (1.00, 1.05) | 0.08 |
| CCI | 1-unit increase | | 0.88 (0.68, 1.14) | 0.33 |

**Regression Models Formulas:**

The final logistic regression model for predicting 3-month retention is represented by

g 1 (x) = -4.6651 + 2.9662*Stool + 0.0330*Age + 0.7951*Educ1 + 2.0381*Educ2

where stool represents the completion of baseline stool sample (1=Yes, 0=No), age is the

patient’s age at enrollment, Educ1 is the attainment of an Associate’s degree/vocational

school/some college (1=Yes, 0=No), and Educ2 is the attainment of Bachelor’s degree or higher

(1=Yes, 0=No). If a participant has only high school education or less, then Educ1=0 and

Educ2=0.

Similarly, the final logistic regression model for predicting 12-month retention is represented by

g 2 (x) = -2.3877 + 1.9937*Stool - 0.7948*Blood + 0.0223*Age + 0.1309*Educ1 + 1.0936*Educ2 - 0.1275*CCI + 0.3239*RAC1 - 0.0716*RAC2

where stool represents the completion of baseline stool sample (1=Yes, 0=No), blood represents

the completion of baseline blood sample (1 = Yes, 0 = No), age is the patient’s age at enrollment,

Educ1 is the attainment of an Associate’s degree/vocational school/some college (1=Yes, 0=No),

Educ2 is the attainment of Bachelor’s degree or higher (1=Yes, 0=No), CCI represents the

patient’s Charlson Comorbidity Index, and RAC1 and RAC2 represent moderately severe or

severe Revised Atlanta Classification for AP, respectively ((1=Yes, 0=No). If a participant has

mild AP, then RAC1=0 and RAC2=0.

To calculate the odds of 3-month retention for a patient who is 30 years old, did not complete

their baseline stool sample (Stool = 0), and has a Bachelor’s degree or higher (Educ2 = 1), these

values can be used in g 1 (x) and then exponentiated to obtain the odds of 3-month retention:

= exp(-4.6651 + 2.9662*0 + 0.0330*30 + 0.7951*0 + 2.0381*1)

The odds of 3-month retention for a patient with these baseline demographics is 0.19. To

compare these odds to a patient who did complete their baseline stool sample, recalculate using

Stool = 1 and divide the two odds to calculate the odds ratio.
